# A Simple Colorimetric Assay for Specific Detection of Glutathione-S Transferase Activity Associated with DDT Resistance in Mosquitoes

**DOI:** 10.1371/journal.pntd.0000808

**Published:** 2010-08-31

**Authors:** Evangelia Morou, Andrew J. Dowd, Shavanti Rajatileka, Andrew Steven, Janet Hemingway, Hilary Ranson, Mark Paine, John Vontas

**Affiliations:** 1 Vector Group, Liverpool School of Tropical Medicine, Liverpool, United Kingdom; 2 Department of Biology, University of Crete, Heraklio, Greece; Mahidol University, Thailand

## Abstract

**Background:**

Insecticide-based methods represent the most effective means of blocking the transmission of vector borne diseases. However, insecticide resistance poses a serious threat and there is a need for tools, such as diagnostic tests for resistance detection, that will improve the sustainability of control interventions. The development of such tools for metabolism-based resistance in mosquito vectors lags behind those for target site resistance mutations.

**Methodology/Principal Findings:**

We have developed and validated a simple colorimetric assay for the detection of Epsilon class Glutathione transferases (GST)-based DDT resistance in mosquito species, such as *Aedes aegypti*, the major vector of dengue and yellow fever worldwide. The colorimetric assay is based on the specific alkyl transferase activity of Epsilon GSTs for the haloalkene substrate iodoethane, which produces a dark blue colour highly correlated with AaGSTE2-2-overexpression in individual mosquitoes. The colour can be measured visually and spectrophotometrically.

**Conclusions/Significance:**

The novel assay is substantially more sensitive compared to the gold standard CDNB assay and allows the discrimination of moderate resistance phenotypes. We anticipate that it will have direct application in routine vector monitoring as a resistance indicator and possibly an important impact on disease vector control.

## Author Summary

*Aedes* mosquitoes transmit many human viral pathogens including dengue, yellow fever and chikungunya. Most of these pathogens have no specific treatment or vaccine and hence their control is reliant on controlling the mosquito vectors, which usually involves the use of insecticides. In order to prevent the alarming prospect of mosquito control failure due to the rapid selection and spread of insecticide resistance in several mosquito populations worldwide, it is essential that effective resistance management strategies are implemented and adhered to. The development of simple diagnostic tests for the early identification and monitoring of resistance is an important prerequisite for this task. Here, we describe the development of a simple colorimetric test for the detection of GSTE2-2/DDTase-based resistance in individual mosquitoes. The novel assay combines the most desirable features of specificity and sensitivity with the low cost and ease of use required for a routine test in endemic countries. It can have direct application in routine vector monitoring as a resistance indicator and help improve the sustainability of insecticide based control strategies.

## Introduction

Prevention of mosquito-borne diseases depends in large part on vector control and usually involves the use of insecticides. Insecticide-based methods include insecticide-impregnated bed nets, indoor or aerial sprays and water treatments. Pyrethroids and the organochlorinated insecticide DDT (1,1,1-dichloro-2,2-bis(p-chlorophenyl)ethylene) are the preferred choice for Indoor Residual Spraying (IRS) and have been used extensively for many decades for the control of disease vectors. Despite environmental concerns, DDT remains one of the cheapest and most effective long-term weapons against vector borne diseases in several stable endemic areas [1]. Although wide scale insecticide implementation has led to impressive decreases in vector borne disease transmission, the emergence and spread of insecticide resistance poses a serious threat and there is a need for new tools that will improve the sustainability of current control interventions [2]. Understanding resistance mechanisms and developing simple diagnostic tests for the early detection of insecticide resistance is an important prerequisite for the application of resistance management strategies.

Insecticide resistance in disease vectors has been attributed to increased rates of insecticide detoxification or mutations in the target sites [3]. Increased rates of glutathione transferase (GST) - mediated DDT dehydrochlorination confers resistance to DDT in several mosquito species, such as *Aedes aegypti*, the major vector of dengue and yellow fever worldwide, and *Anopheles gambiae*, the major malaria vector in sub-saharan Africa [4], [5]. This DDT detoxification reaction is catalysed by the Epsilon class GST, GSTE2-2 in *An. gambiae*, *An. cracens* and *Ae. aegypti* mosquitoes from different geographical origins [4], [5], [6].

Detection of metabolism – based insecticide resistance is more complex than screening for specific mutations known to cause target site resistance. Current techniques for measuring elevated GSTE2-2 levels in mosquitoes, such as real time PCR or specific ELISA based on antibodies are elaborate or require the use of expensive equipment and consumables, and are therefore not accessible to laboratories on a limited budget [4], [5].

Biochemical assays for detecting metabolic resistance generally employ generic substrates that are recognised by most or all members of the enzyme families. For example, GST activity is usually measured using 1-chloro-2,4-dinitrobenzene (CDNB), 1,2-dichloro-4-nitrobenzene (DCNB), and, more recently, monoclorobimane [7], [8]. Unlike assays to detect elevated esterase activity, which can be read by eye, the current GST assays require a spectrophotometer that can measure absorbance in the UV range, or fluorimeter with multiple emission/excitation channels [8], [9], limiting their applicability in the field. Potentially greater sensitivity and specificity could be achieved if substrates that were specifically recognised by the enzyme(s) responsible for insecticide metabolism were employed.

A colorimetric assay for GSTs with alkyl transferase activity, capable of catalysing the release of iodine from haloalkene substrates, has been recently described [10]. Using a modified version of this assay, Dowd et al. [11] screened a large number of recombinant mosquito GSTs for alkyltransferase activity with several haloalkene substrates, to identify potential enzyme biosensors for detecting insecticides. Recombinant epsilon GSTs, but not the delta or sigma GSTs, which are the most abundant in insects [12], showed a remarkable ability to utilise iodoethane as a substrate and produce a dark blue colour, which can be measured spectrophotometrically or visually [11].

Here, we have adapted the alkyl transferase/iodoethane -based colorimetric assay to measure GST activity associated with DDT resistance in individual *Ae. aegypti* mosquitoes.

## Materials and Methods

### Mosquito strains and bioassays

Six *Ae. aegypti* mosquito strains were used in this study: The standard laboratory reference strain (New Orleans) was kindly provided by the Center for Disease Control and Prevention (CDC), Atlanta, USA, the susceptible Ivory Coast strain was collected from Cote d'Ivoire, the Iquitos strain originating from Peru and the Solidaridad, Isla Mujeres and Merida strains, from Mexico, were kindly provided by Prof. William Black (Colorado State University, USA). All strains were reared under standard conditions (28°C±2°C, 80% RH) at the Liverpool School of Tropical Medicine. Bioassays were performed on 1–3 day old adults using the World Health Organization (WHO) adult susceptibility test papers – DDT 4% [7]. The time causing 50% mortality (LT50) was obtained 24h after the exposure.

### Cloning, expression and purification of mosquito GSTs

Cloning into a pET3a vector, expression in *Escherichia coli* BL21(DE3) plysS, and purification of *Ae. aegypti* recombinant Epsilon GSTs were conducted as described previously [5], [13]. The eluted enzyme was concentrated using a Vivaspin 15R concentrator and exchanged using a PD-10 column into 50 mM sodium potassium phosphate (pH 7.4), 10 mM dithiothreitol, and 40% glycerol according to the manufacturer's instructions and samples were stored at −80°C, until used.

### Biochemical assays

Mosquitoes were homogenised in 0.1M Tris-HCl, pH 8.2 (20 µl per individual), the mixture was centrifuged at 14,000×g for 10 min at 4°C, and the supernatant was used as the enzyme source for the biochemical assays. Standard GST spectrophotometric assays were performed by monitoring the formation of the conjugate of CDNB or 1, 2-dichloro-4-nitrobenzene (DCNB), and reduced glutathione (GSH) [9]. The iodide-releasing reaction was carried out as previously described [10] and optimised by Dowd et al. [11], with GSH (2.5 mM) and iodoethane (2.5 mM) in 0.1M phosphate buffer pH 8.2 and enzyme source in a total volume of 100 µl at 25°C. The reaction was incubated at 30 min, or for different periods of time depending on the reaction rate studied during optimisation stages. Blue colour developed immediately after addition of 50 µl starch solution (0.25 g partially hydrolysed potato starch in 25 ml of Milli-Qwater and boiled in a microwave oven until all starch has dissolved) and 100 µl acidified peroxide solution (2% H_2_O_2_ in 2 mM HCl). The blue colour was quantified spectrophotometrically at 610 nm using a VERSAmaxTM microplate spectrophotometer (Molecular Devices, Sunnyvale, CA, USA), or estimated visually by eye. A standard curve was prepared from different concentrations of KI in 0.1M Tris–HCl buffer, pH 8.2. Specific activities towards iodoethane were calculated from the linear range of the enzymatic reaction, and a plot of absorbance at 610 nm against potassium iodide concentration. They are expressed as µmole of iodide released /min/mg. All measurements were made in triplicate. Protein concentrations were measured using Bio-Rad protein assay reagent with bovine serum albumin as the protein standard [14].

### Western blot analysis

Mosquito extracts (0.060 mg total protein) were analysed with SDS-polyacrylamide gel electrophoresis (10% acrylamide running gel and 4% acrylamide stacking gel) and electroblotted onto polyvinylidene difluoride membrane. The membrane was probed for 2 hours with an anti-AaGSTE2-2 antibody at 1∶1000 dilution in 3% milk-PBS-Tween solution and for 1 hour with a peroxidase-labelled anti-rabbit antibody at 1∶10000 dilution. Immunoreactive proteins were visualised using a horseradish peroxidase sensitive ECL chemiluminescent Western blotting kit (GE Healthcare).

## Results

### Optimisation of the alkyltransferase/iodoethane assay for measuring GST activity colorimetrically in individual mosquitoes

**Figure 1:**
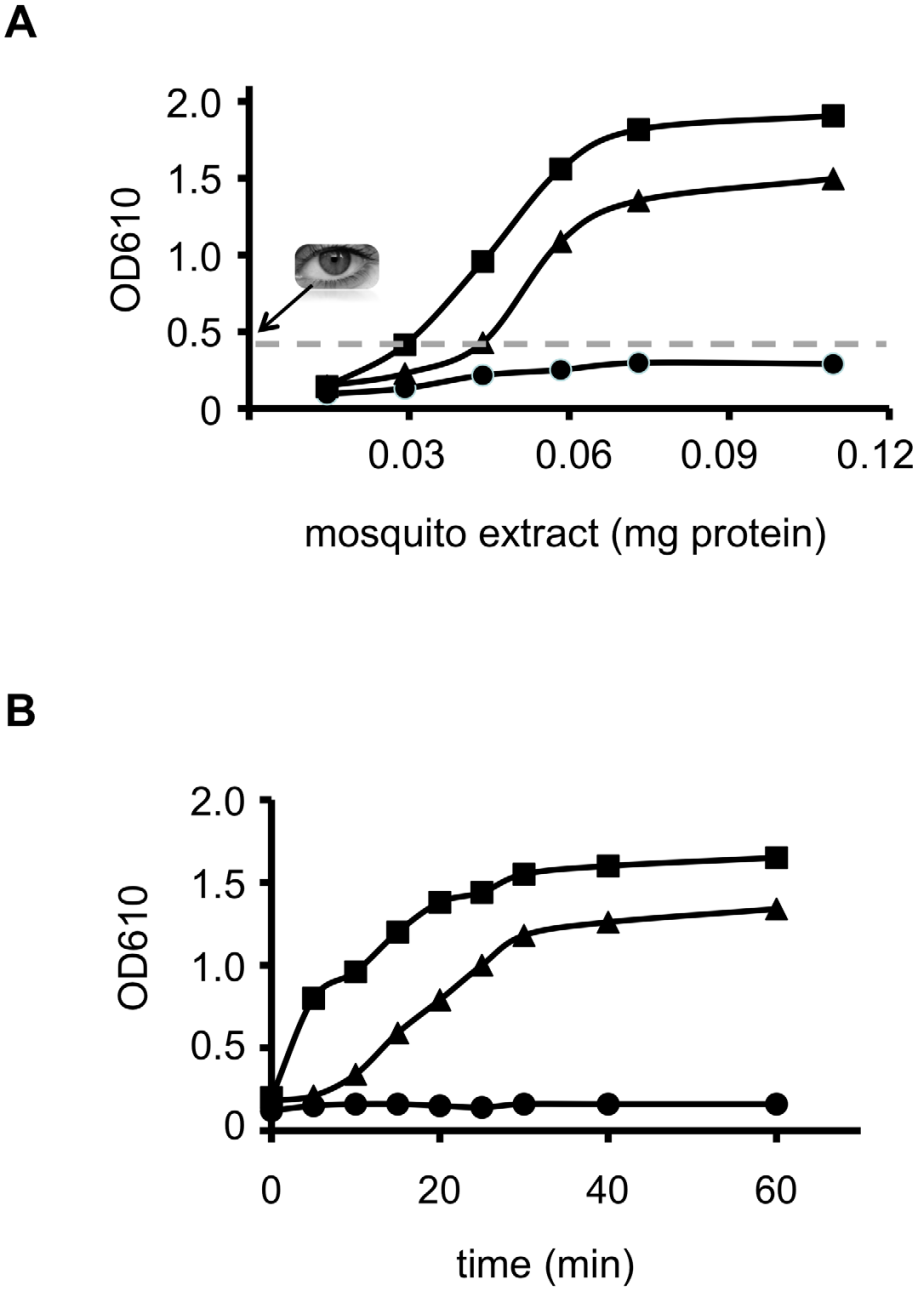
Effect of *Aedes aegypti* crude homogenate concentration and incubation time on enzyme product/colour formation. (A) Effect of homogenate concentration on product/colour formation. Various amounts of extract from three different mosquito strains were incubated in the reaction mixture with 2.5 mM iodoethane for 30 min, the reaction was subsequently terminated and colour development was recorded spectrophotometrically. Eye and the dotted line signify the OD threshold that can be easily visualised by eye. Each value represents the mean of 5 independent determinations. The SD was below 5% for all mosquito protein extracts tested. (B) Time course of product/colour formation: 0.060 mg mosquito homogenate (equivalent to approximately ¾ of an individual *Ae. aegypti* female) was incubated with 2.5 mM iodoethane at various time points from 5 to 60 min before terminating the reaction and recording colour development. New Orleans (reference) strain is indicated with a circle, while the Solidaridad and Merida strains are depicted with a trangle and a square, respectively. Each value represents the mean of 5 independent determinations. The SD was below 5% for all time points.

We recently showed that, unlike other GST members tested, epsilon GSTs can very efficiently utilise the haloalkene iodoethane as a substrate [11]. In order to determine the amount of mosquito protein required to measure GST activity in the visual range (colour change) and set the linear limits of the colorimetric assay, we tested different amounts of mosquito extracts (0.010–0.120 mg of total protein) at time points between 5 and 60 min. The minimum amount of protein extract that gave a visible colour range in any mosquito strain, after 30min incubation period, was 0.030 mg (Figure 1A). No visible colour change was observed for the reference susceptible strain New Orleans, even when much higher amounts of protein (and longer incubation times up to 60 min, data not shown) were included in the assay (Figure 1A).

The product/colour formation is linear for at least 30 min, when approximately 0.060 mg mosquito homogenate (equivalent to ¾ of an individual *Ae. aegypti* female) was assayed (Figure 1B). The linear range of the reaction was not affected by temperature fluctuations between 25 and 35°C (data not shown).

### Utility of the alkyltransferase/iodoethane assay for the specific detection of GST-based insecticide resistance in individual mosquitoes

**Figure 2:**
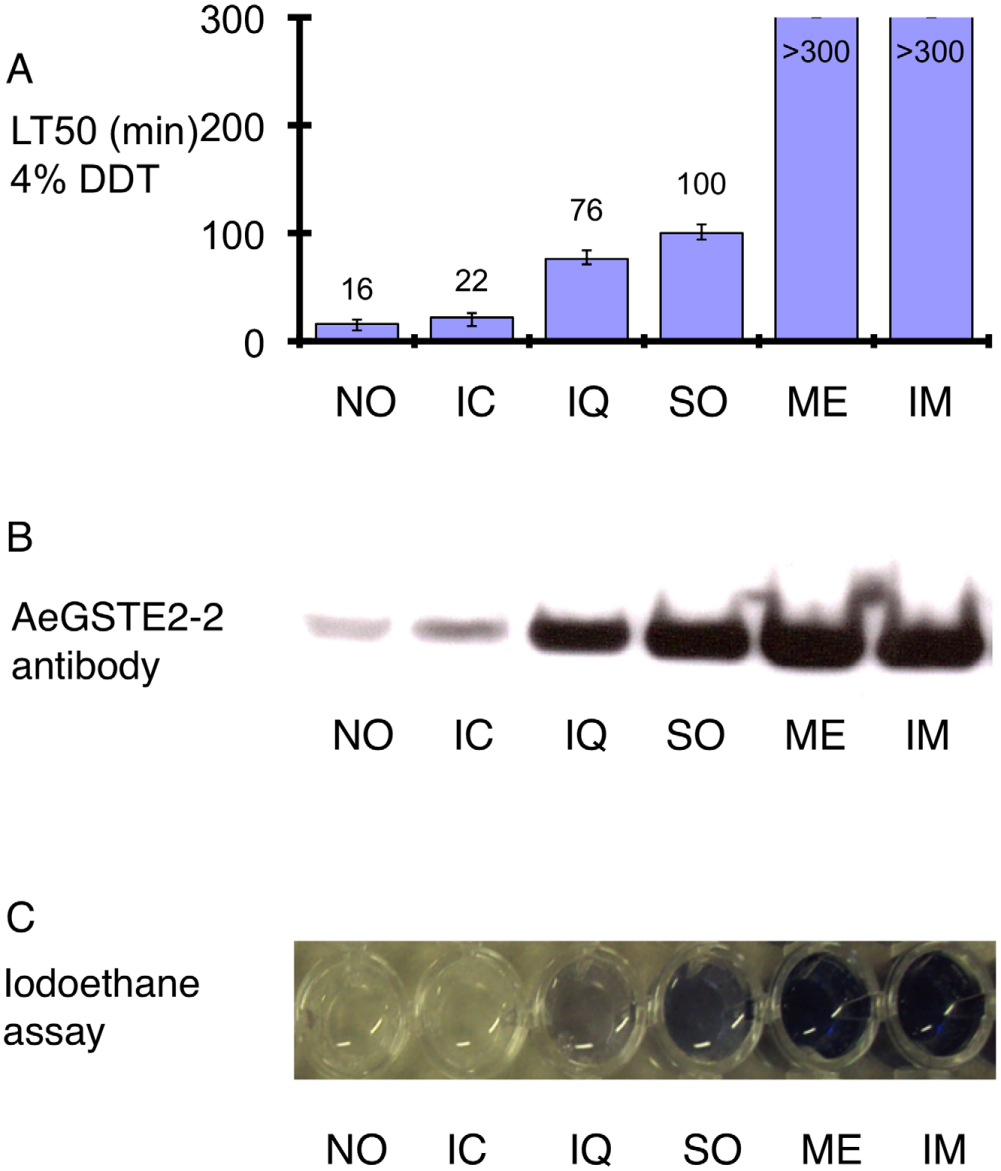
Determination of the effectiveness of the alkyltransferase/iodoethane assay for the specific detection of epsilon GST-based insecticide resistance in individual *Aedes aegypti* mosquitoes. (A) The lethal time for 50% mortality (LT50, the time causing 50% mortality) obtained 24h after exposing adult females of several *Aedes aegypti* strains to 4% DDT, using WHO adult susceptibility test papers. NO: N. Orleans, IC: Ivory Coast, IQ: Iquitos, SO: Solidaridad, ME: Merida and IM: Isla Mujeres strains. (B) Crude homogenates (µg) from *Ae. aegypti* strains with variable DDT resistance levels were resolved by SDS/PAGE on 10 polyacrylamide gels. Approximately 0.060 mg of protein was loaded into each lane. The proteins from the SDS/PAGE gel were transferred to a nitrocellulose membrane and probed with antisera raised against AaGSTE2-2. Strain abbreviations as in Figure 2A. (C) Microtiter plate demonstrating the colour formation of crude homogenates from single mosquitoes from susceptible (NO and IC), intermediate resistant (IQ and SO) and resistant (ME and IM) strains. Strain abbreviations as in Figure 2A.

The LT50 values of six *Ae. aegypti* mosquito strains following exposure in 4% DDT were determined (Figure 2A). The susceptible New Orleans and Ivory Coast strains showed LT50 values of 20 min or less, whilst the Iquitos and Solidaridad strains exhibited LT50 values of 76 min and 100 min, respectively; accurate LT50 values could not be determined for Merida and Isla Mujeres strains, due to the very high levels of resistance (LT50>300 min). To confirm the association of the AaGSTE2-2 enzyme with the resistance phenotype, we performed Western blot analysis, using crude mosquito homogenates probed with anti-AaGSTE2-2 antiserum. A single band of approximately 25 kDa was detected in all strains, with intensity levels highly correlated with the LT50 values/DDT resistance data (Figure 2B).

Using the optimised colorimetric assay, we determined the specific GST activity in adult females from several *Ae. aegypti* strains. As shown in Table 1, there is a >15-fold difference in alkyl transferase activity between the highly DDT resistant Merida and Isla Mujeres strains, and the susceptible Ivory Coast strain. The alkyl transferase activity of the Iquitos and Solidaridad strains, which showed moderate resistance levels, was 4- and 12-fold higher, respectively, compared with the Ivory Coast strain (Table 1). A highly significant correlation was observed between the LT50s and the enzymatic activities obtained by the iodoethane/colorimetric assay (R^2^ = 0.97, P<0.01). The difference in alkyltransferase activity between the different strains can be easily visualised by eye (Figure 2C), via the effort of multiple individuals.

**Table 1:** Comparison of the GST specific activity among several *Aedes aegypti* mosquito strains with various resistance levels to DDT, using the substrates CDNB, DCNB and Iodoethane.

Mosquito strains	LT50s (min)	Iodoethane	CDNB	DCNB
Ivory Coast	20	0.014±0.003^a^	0.6±0.2^a^	0.03±0.01^b^
New Orleans	16	0.020±0.003^a^	0.9±0.2^a^	0.09±0.02^a^
Iquitos	76	0.060±0.002^b^	2.4±0.4^b^	0.19±0.05^c^
Solidaridad	100	0.170±0.015^c^	2.6±0.3^b^	0.13±0.03^a^
Merida	>300	0.320±0.025^d^	2.6±0.3^b^	0.15±0.02^c^
Isla Mujeres	>300	0.290±0.020^d^	2.9±0.5^b^	0.15±0.06^c^

Three assays were performed from *n* = 10 mosquitoes, collected from 3 independent biological replicates. Values are the means S.E.M. Specific activities are given in µmole iodide released/min/mg for the iodoethane assay and µmole/min/mg for the CDNB and DCNB assays (units/mg of protein). In the same column, values with different superscript letters are significantly different (P<0.01).

This correlation between resistance phenotype and specific activity does not hold for the model substrates CDNB and DCNB. For CDNB there was significantly higher activity in the four resistant strains compared to the two susceptible stains (Table 1) but no difference in activity between the moderately and highly resistant groups. For DCNB, the relationship was even less clear. For example no significant difference was observed between the moderate resistant strain Solidaridad and the New Orleans susceptible strain (Table 1).

### Specificity of alkyltransferase/iodoethane colorimetric assay for GSTE2-2/DDTase activity

By screening a large number of recombinant mosquito GSTs for alkyltransferase activity with several substrates, Dowd et al. [11] showed that mosquito epsilon GSTs, AaGSTE2-2 and AaGSTE4-4, can utilise the haloalkene iodoethane as substrate but that this substrate was not recognised by delta or sigma class GSTs. To determine whether the ability to catalyse the release of iodine from iodoethane was a general property of epsilon GSTs, we expressed six family members and measured their specific activity against this substrate. As shown in Table 2, the highest activity was obtained with the DDTase AaGSTE2-2 (10.3 µmole iodide/min/mg). Other members of the Epsilon class also recognised this substrate but their specific activities were lower (0.03 to 4.3 µmole iodide/min/mg). AaGSTE8-8 exhibited the lowest activity, possibly due to the low amino acid identity (approximately 30%) of this gene with other members of this class [15]. The respective CDNB activities of the recombinant epsilon GSTs are also shown in Table 2 for comparison. The DDTase AaGSTE2-2 has lower or similar specific activity with CDNB compared to other members of the family and hence this substrate cannot specifically recognise GSTs implicated in insecticide resistance.

**Table 2:** Specific activities of recombinant epsilon GST enzymes determined with iodoethane or CDNB substrates.

Recombinant GST	Accession number	CDNB activity	Iodoethane activity
AaGSTE2-2	AAEL007951	5±0.3	10.3±0.87
AaGSTE4-4	AAEL007962	10.2±0.4	4.3±0.20
AaGSTE3-3	AAEL007947	20.8±0.7	3.2±0.10
AaGSTE7-7	AAEL007948	2.6±0.1	1.1±0.10
AaGSTE5-5	AAEL007964	7.4±0.2	0.7±0.10
AaGSTE8-8	AAEL007955	4.7±0.1	0.03±0.03

Specific activities as in Table 1. GSTs are ranked according to iodoethane activity. Results show mean value from n = 3 independent replicates.

## Discussion

We have developed a simple colorimetric assay for the specific detection of GST activity associated with DDT resistance in Ae. aegypti. The colorimetric assay is substantially more sensitive in detecting DDT resistance in *Ae. aegypti*, compared to the gold standard CDNB assay currently being used in routine mosquito resistance monitoring studies [7]. The differences in GST activities among strains with high, moderate or negligible resistance were over 15-fold for iodoethane, but only 1.5–4.3-fold for CDNB and DCNB substrates. In contrast to iodoethane, the latter general substrates failed to discriminate moderate resistance phenotypes (Table 1). This increased sensitivity of the novel colorimetric assay provides greater potential for the identification of resistance at early stages, a crucial pre-requisite for the implementation of evidence-based resistance management tactics.

Unlike UV/spectrophotometric CDNB and DCNB assays, the alkyltransferase/iodoethane assay produces a dark blue colour that is both highly correlated with AaGSTE2-2-overexpression-based DDT resistance and can be estimated by eye at least semi-quantitatively (Figure 2). This novel assay can be performed by non-qualified personnel, without sophisticated equipment. It is robust at temperatures between 25–35°C, with a wide linear range of quantification, and a sensitivity which allows the measurement of GST activity in a single mosquito. The cost of the assay is less than 0.05 USD per mosquito, while the shelf life of the substrate iodoethane is at least 1 year at 4°C.

Here, we have focused on *Ae. aegypti*, as DDT resistance is extremely high in many populations of this species in dengue endemic regions [16]. However, the assay can be adapted for measuring GSTE2-2/DDTase – based DDT resistance in other mosquito species, such as the major malaria vector *An. gambiae*. This was not tested here, as there were no suitable resistant strains available. Nevertheless, given that iodoethane is a very good substrate also for the orthologue enzyme AgGSTE2-2 (data not shown) and this enzyme is the key enzyme responsible for DDT resistance in this species [4], there is no reason to believe that this assay will not work for *Anopheles* mosquitoes too.

In conclusion, we describe a simple colorimetric test for the detection of the GSTE2-2/DDTase- based resistance in mosquitoes. It combines the most desirable features of specificity and sensitivity with the low cost and ease of use required for a routine test in endemic countries. We anticipate that the assay will have direct application in routine vector monitoring as a resistance indicator and help improve the sustainability of insecticide based control strategies.
